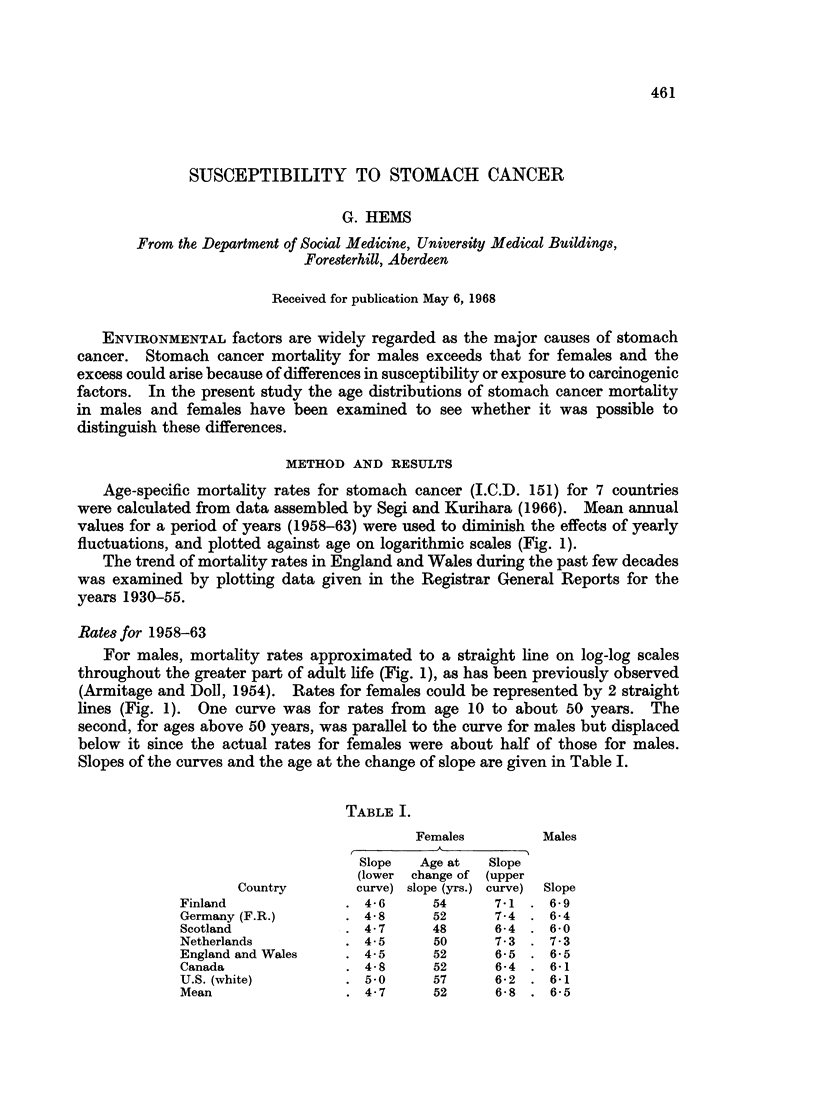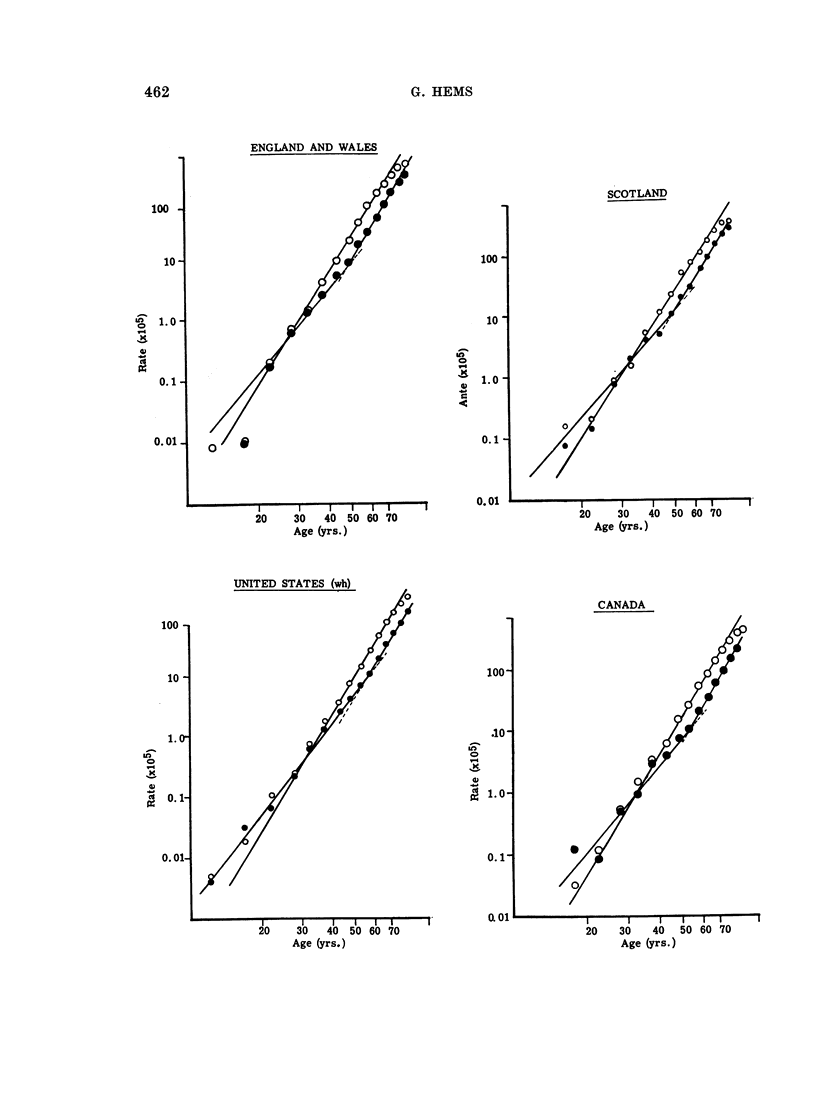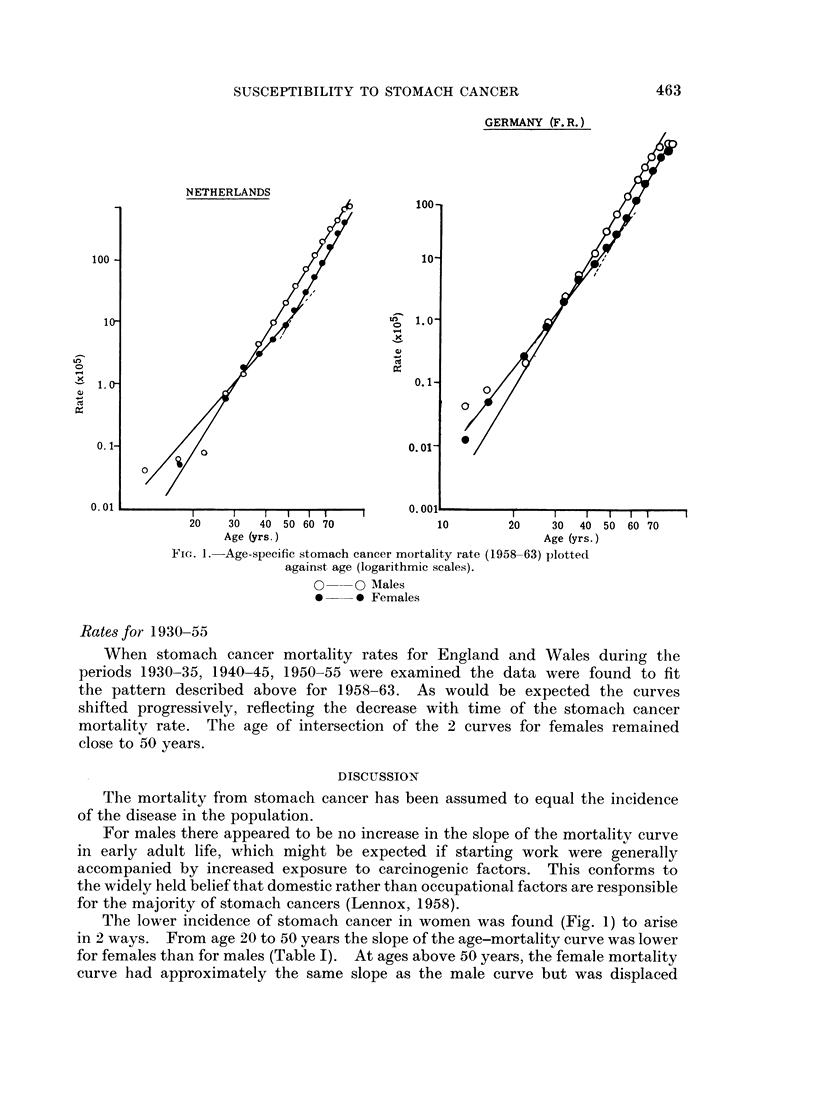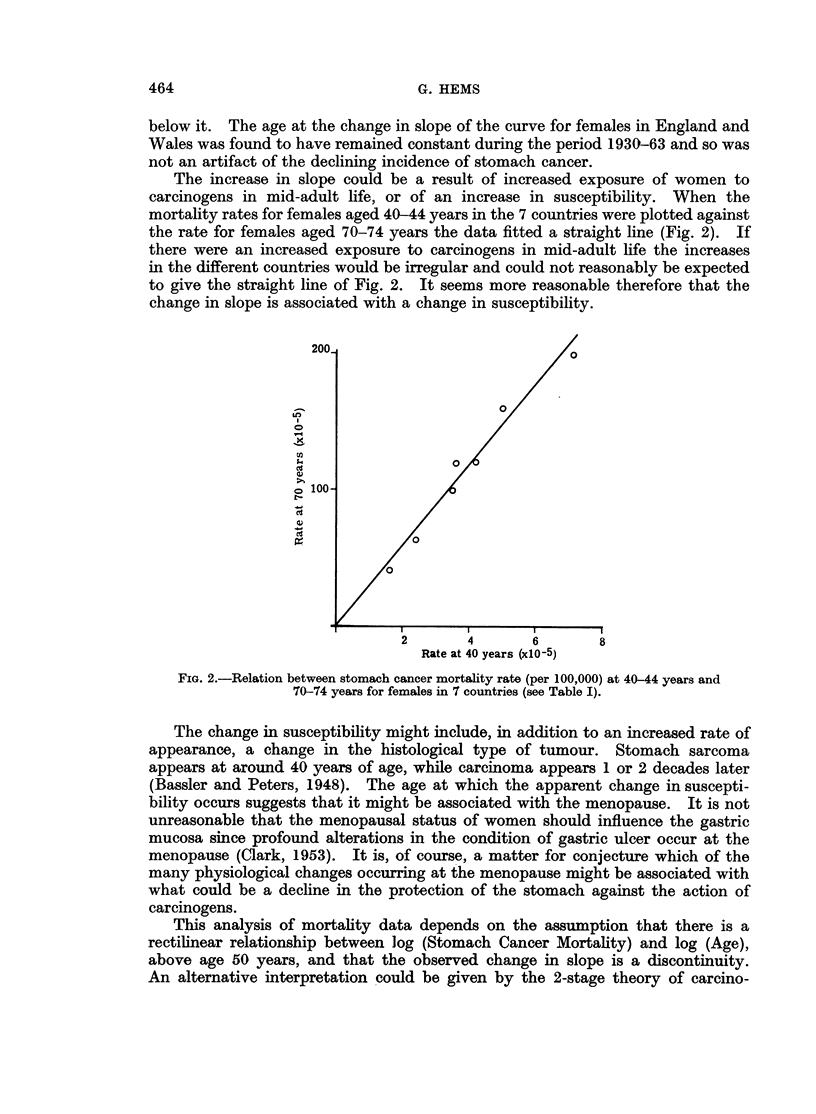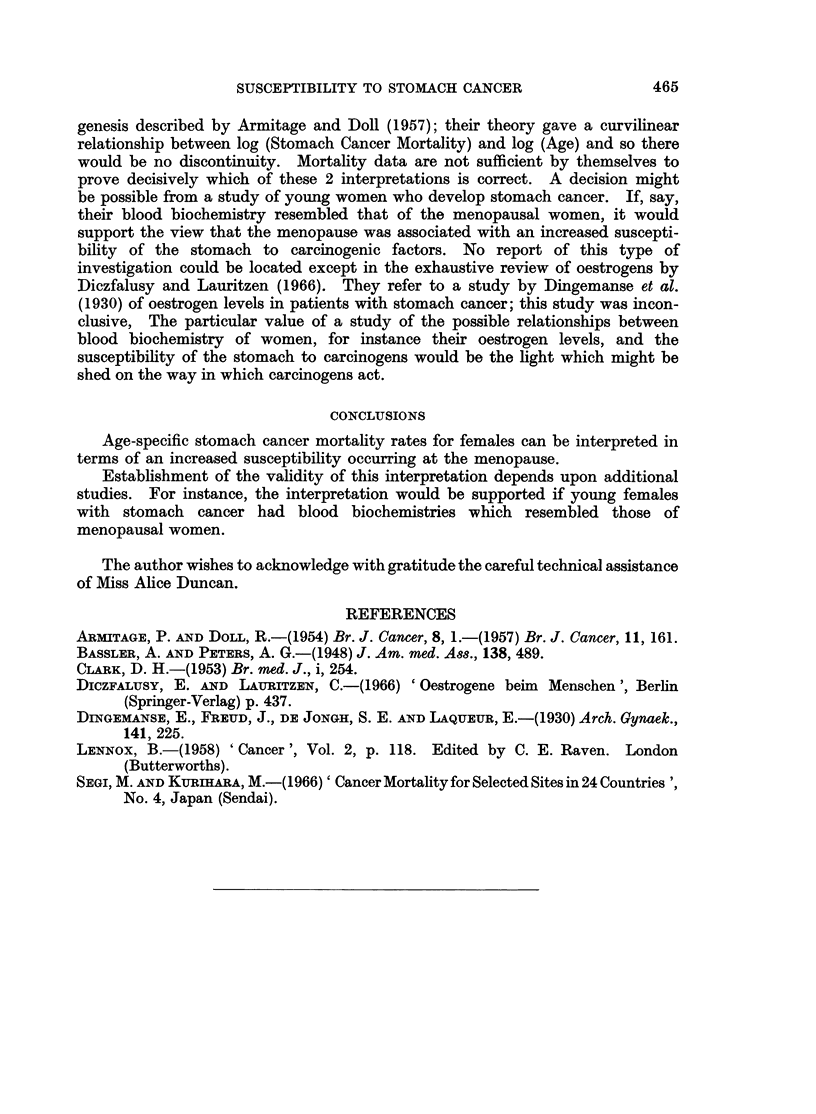# Susceptibility to stomach cancer.

**DOI:** 10.1038/bjc.1968.53

**Published:** 1968-09

**Authors:** G. Hems


### SUSCEPTIBILITY TO STOMACH CANCER

G. HEMS

*From the Department of Social Medicine, University Medical Buildings, Foresterhill, Aberdeen*

Received for publication May 6, 1968

ENVIRONMENTAL factors are widely regarded as the major causes of stomach cancer. Stomach cancer mortality for males exceeds that for females and the excess could arise because of differences in susceptibility or exposure to carcinogenic factors. In the present study the age distributions of stomach cancer mortality in males and females have been examined to see whether it was possible to distinguish these differences.

### METHOD AND RESULTS

Age-specific mortality rates for stomach cancer (I.C.D. 151) for 7 countries were calculated from data assembled by Segi and Kurihara (1966). Mean annual values for a period of years (1958–63) were used to diminish the effects of yearly fluctuations, and plotted against age on logarithmic scales (Fig. 1).

The trend of mortality rates in England and Wales during the past few decades was examined by plotting data given in the Registrar General Reports for the years 1930–55.

### *Rates for* 1958–63

For males, mortality rates approximated to a straight line on log-log scales throughout the greater part of adult life (Fig. 1), as has been previously observed (Armitage and Doll, 1954). Rates for females could be represented by 2 straight lines (Fig. 1). One curve was for rates from age 10 to about 50 years. The second, for ages above 50 years, was parallel to the curve for males but displaced below it since the actual rates for females were about half of those for males. Slopes of the curves and the age at the change of slope are given in Table I.

TABLE I.

| | Females | | | Males |
|---|---|---|---|---|
| Country | Slope (lower curve) | Age at change of slope (yrs.) | Slope (upper curve) | Slope |
| Finland | 4·6 | 54 | 7·1 | 6·9 |
| Germany (F.R.) | 4·8 | 52 | 7·4 | 6·4 |
| Scotland | 4·7 | 48 | 6·4 | 6·0 |
| Netherlands | 4·5 | 50 | 7·3 | 7·3 |
| England and Wales | 4·5 | 52 | 6·5 | 6·5 |
| Canada | 4·8 | 52 | 6·4 | 6·1 |
| U.S. (white) | 5·0 | 57 | 6·2 | 6·1 |
| Mean | 4·7 | 52 | 6·8 | 6·5 |

ENGLAND AND WALES

Rate (x10^5)

100

10

1.0

0.1

0.01

20

30

40

50

60

70

Age (yrs.)

SCOTLAND

Ante (x10^5)

100

10

1.0

0.1

0.01

20

30

40

50

60

70

Age (yrs.)

UNITED STATES (wh)

Rate (x10^5)

100

10

1.0

0.1

0.01

20

30

40

50

60

70

Age (yrs.)

CANADA

Rate (x10^5)

100

.10

1.0

0.1

0.01

20

30

40

50

60

70

Age (yrs.)

FIG. 1.—Age-specific stomach cancer mortality rate (1958–63) plotted against age (logarithmic scales).

○——○ Males
●——● Females

*Rates for* 1930–55

When stomach cancer mortality rates for England and Wales during the periods 1930–35, 1940–45, 1950–55 were examined the data were found to fit the pattern described above for 1958–63. As would be expected the curves shifted progressively, reflecting the decrease with time of the stomach cancer mortality rate. The age of intersection of the 2 curves for females remained close to 50 years.

### DISCUSSION

The mortality from stomach cancer has been assumed to equal the incidence of the disease in the population.

For males there appeared to be no increase in the slope of the mortality curve in early adult life, which might be expected if starting work were generally accompanied by increased exposure to carcinogenic factors. This conforms to the widely held belief that domestic rather than occupational factors are responsible for the majority of stomach cancers (Lennox, 1958).

The lower incidence of stomach cancer in women was found (Fig. 1) to arise in 2 ways. From age 20 to 50 years the slope of the age–mortality curve was lower for females than for males (Table I). At ages above 50 years, the female mortality curve had approximately the same slope as the male curve but was displaced below it. The age at the change in slope of the curve for females in England and Wales was found to have remained constant during the period 1930–63 and so was not an artifact of the declining incidence of stomach cancer.

The increase in slope could be a result of increased exposure of women to carcinogens in mid-adult life, or of an increase in susceptibility. When the mortality rates for females aged 40–44 years in the 7 countries were plotted against the rate for females aged 70–74 years the data fitted a straight line (Fig. 2). If there were an increased exposure to carcinogens in mid-adult life the increases in the different countries would be irregular and could not reasonably be expected to give the straight line of Fig. 2. It seems more reasonable therefore that the change in slope is associated with a change in susceptibility.

FIG. 2.—Relation between stomach cancer mortality rate (per 100,000) at 40–44 years and 70–74 years for females in 7 countries (see Table I).

The change in susceptibility might include, in addition to an increased rate of appearance, a change in the histological type of tumour. Stomach sarcoma appears at around 40 years of age, while carcinoma appears 1 or 2 decades later (Bassler and Peters, 1948). The age at which the apparent change in susceptibility occurs suggests that it might be associated with the menopause. It is not unreasonable that the menopausal status of women should influence the gastric mucosa since profound alterations in the condition of gastric ulcer occur at the menopause (Clark, 1953). It is, of course, a matter for conjecture which of the many physiological changes occurring at the menopause might be associated with what could be a decline in the protection of the stomach against the action of carcinogens.

This analysis of mortality data depends on the assumption that there is a rectilinear relationship between log (Stomach Cancer Mortality) and log (Age), above age 50 years, and that the observed change in slope is a discontinuity. An alternative interpretation could be given by the 2-stage theory of carcinogenesis described by Armitage and Doll (1957); their theory gave a curvilinear relationship between log (Stomach Cancer Mortality) and log (Age) and so there would be no discontinuity. Mortality data are not sufficient by themselves to prove decisively which of these 2 interpretations is correct. A decision might be possible from a study of young women who develop stomach cancer. If, say, their blood biochemistry resembled that of the menopausal women, it would support the view that the menopause was associated with an increased susceptibility of the stomach to carcinogenic factors. No report of this type of investigation could be located except in the exhaustive review of oestrogens by Diczfalusy and Lauritzen (1966). They refer to a study by Dingemanse *et al.* (1930) of oestrogen levels in patients with stomach cancer; this study was inconclusive, The particular value of a study of the possible relationships between blood biochemistry of women, for instance their oestrogen levels, and the susceptibility of the stomach to carcinogens would be the light which might be shed on the way in which carcinogens act.

### CONCLUSIONS

Age-specific stomach cancer mortality rates for females can be interpreted in terms of an increased susceptibility occurring at the menopause.

Establishment of the validity of this interpretation depends upon additional studies. For instance, the interpretation would be supported if young females with stomach cancer had blood biochemistries which resembled those of menopausal women.

The author wishes to acknowledge with gratitude the careful technical assistance of Miss Alice Duncan.

### REFERENCES

ARMITAGE, P. AND DOLL, R.—(1954) *Br. J. Cancer*, **8**, 1.—(1957) *Br. J. Cancer*, **11**, 161.

BASSLER, A. AND PETERS, A. G.—(1948) *J. Am. med. Ass.*, **138**, 489.

CLARK, D. H.—(1953) *Br. med. J.*, i, 254.

DICZFALUSY, E. AND LAURITZEN, C.—(1966) ‘Oestrogene beim Menschen’, Berlin (Springer-Verlag) p. 437.

DINGEMANSE, E., FREUD, J., DE JONGH, S. E. AND LAQUEUR, E.—(1930) *Arch. Gynaek.*, **141**, 225.

LENNOX, B.—(1958) ‘Cancer’, Vol. 2, p. 118. Edited by C. E. Raven. London (Butterworths).

SEGI, M. AND KURIHARA, M.—(1966) ‘Cancer Mortality for Selected Sites in 24 Countries’, No. 4, Japan (Sendai).